# Molecular mechanism of ocular surface damage: Application to an in vitro dry eye model on human corneal epithelium

**Published:** 2011-01-12

**Authors:** Marisa Meloni, Barbara De Servi, Daniela Marasco, Salvatore Del Prete

**Affiliations:** 1 VitroScreen-In Vitro Research Laboratories, Milano, Italy; 2 CISME (Centro Interdipartimentale di Microscopia Elettronica) Università di Napoli Federico II, Napoli, Italy

## Abstract

**Purpose:**

The present study was concerned with the development of a new experimental model of dry eye using human reconstructed in vitro corneal epithelium (HCE). The model is based on the use of adapted culture conditions that induce relevant modifications at the cellular and molecular level thus mimicking dry eye.

**Methods:**

The HCE model was maintained in a controlled environmental setting (relative humidity <40% and 40 °C temperature) for 24 h and up to 72 h to induce dry eye. The evolution of the dry eye condition was assessed by histology, immunohistochemistry staining, scanning electron microscopy, and gene expression by using TaqMan gene assay technology (mucin-4 [*MUC4*], matrix metallopeptidase-9 [*MMP9*], tumor necrosis factor-α [*TNF-α*], and defensin β-2 [*DEFB2*). The effects of different commercially available tear substitutes on the induced dry eye condition were tested.

**Results:**

This in vitro dry eye HCE model, that was well established within 24 h, has the characteristic features of a dry eye epithelium and could be satisfactorily used for preliminary assessment of the protective activity of some artificial tears. The transcriptional study of selected biomarkers showed an increase in *MUC4*, *MMP9*, *TNF-α*, and hBD-2 (*DEFB2*) gene expression.

**Conclusions:**

By using a dynamic approach, we were able to define a biomarker gene signature of dry eye-induced effects that could be predictive of corneal damage in vivo and to discriminate the efficacy among different commercial artificial tears.

## Introduction

Dysfunctional tear syndrome (DTS) or dry eye disease is a multifactorial disease of the tears and ocular surface that results in symptoms of discomfort, modification of the tear film composition, and tear film instability, with potential damage to the ocular surface [1]. The precorneal tear film has a three-layer structure: a mucous layer in contact with the ocular surface, an intermediate aqueous layer, and an anterior lipid layer. The aqueous layer is involved in lubrication and protection of the ocular surface and contains soluble mucin and microvilli. Mucins are large, highly glycosylated glycoproteins that are major components of the mucus layer adjacent to ocular surface epithelia. They play a key role in retention of water and other tear fluid components on the surface of the apical cell, thus preventing desiccation of the epithelial surface [2]. Mucins also lubricate the ocular surface during the eyelid blink, help to maintain a smooth refractive surface, and provide a barrier to pathogen penetration. The epithelial cells contribute to secretion of tear mucus and the microvilli of the apical epithelial cells increase the surface for tear adherence. The protective function of the microvilli (which change in amount and structure) is closely related to tear film stability [3]. In dry eye, dysfunction of the ocular surface-secretory glandular functional unit can lead to an epithelial disease, termed keratoconjunctivitis sicca (KCS) [1]. Dry eye symptoms range from mild, transient irritation to persistent dryness, burning, itchiness, redness, pain, ocular fatigue, and visual disturbance. Dry eye disease is associated with a decrease in aqueous tear production, abnormalities of the lipid, protein, and mucin profiles [4], and an increased osmolarity and inflammation of the ocular surface. Increased concentrations of proinflammatory cytokines have been implicated in dry eye syndrome, which occur either on the ocular surface or in the lacrimal gland. Reduced levels of growth factor and soluble mucin, and the presence of activated proteases (such as matrix metallopeptidase [MMP9] that degreade the extracellular matrix) along with the tight junction protein occludin are also observed [5-8].

Several different animal models of dry eye have been proposed, including surgical removal of the tear-producing glands, ocular surface desiccation by mechanical inhibition of blinking, and pharmacologic inhibition of tear secretion [9]. However, these models are labor-intensive and difficult to manage for prolonged periods. All existing animal models of dry eye mimic different pathogenic mechanisms of KCS, but none appear to mirror precisely the molecular and morphological modification characteristic of a DTS. A dry eye non-animal model that mimics human dry eye disease would be a useful tool to investigate the multiple factors implicated in the pathogenesis of KCS.

The corneal epithelium, a non-keratinized multilayered squamous epithelium of about 60 μm, plays a crucial role in the barrier function and can be considered the first line of defense against many types of injury, trauma, or infection. Furthermore, the corneal epithelium is directly involved in the establishment of dry eye and the discomfort and pain associated with dry eye syndrome. In vitro, 3D models of human corneal epithelium have been developed during the last ten years [10]. These models are versatile for the set-up of modified protocols and allow objective and reproducible quantification of complementary testing parameters [11-14]. Compared to in vivo studies on animals and cell culture models, the in vitro tissue is an easy-to-handle model of human origin that more closely resembles human epithelial physiology than conventional monolayer models. In vitro tissue has increased permeability compared to in vivo tissue [15,16]; however, this could be considered as a further advantage for the design of sensitive experimental models that allow early detection of the effects of sub-toxic doses.

The purpose of this study was to establish experimentally induced dry eye in vitro (EDEV) on a human corneal epithelium (HCE) model. Culture conditions were modified to induce the most relevant morphological, cellular, and molecular modifications related to dry eye symptoms, namely inflammation and modification of the structural compartments of the ocular surface and microvilli ‘network’. By using positive and negative HCE controls in our experimental model, various parameters likely to be modified by specific culture conditions were investigated. Cellular viability was assessed by the Alamar Blue test, epithelium morphology was examined by light microscopy, and the structure of epithelial surface microvilli assessed by scanning electron microscopy (SEM). Transcriptional analysis of a selected gene signature was performed via real-time reverse transcription polymerase chain reaction (qRT–PCR). The genes analyzed included *MMP9* (matrix metallopeptidase-9) and *TNFα* (tumor necrosis factor-α, an inflammatory mediator), as increased expression of both has been observed in ocular surface epithelial diseases and in the tear fluid of patients with dry eye [17-19]. Mucin-4 (*MUC-4*) expression was analyzed due to its known lubricating and clearing function and its role as a surface-associated mucin in providing barrier function to corneal and conjunctival epithelia [20,21]. Expression of human defensin β-2 (*DEFB2*) was evaluated due its role in antimicrobial protection and in ocular surface damage in subjects with dry eye [22,23]. After optimizing experimental conditions for the induction of a dry and inflamed epithelium (Part 1), different commercially available formulations called artificial tears were assessed (Part 2). They do not have a direct pharmacological activity but containing polymers of various origin (biotechnology and natural derived or synthetic) they are able to directly interact with the ocular surface and in particular with the corneal epithelium. In vivo they can show a lubrificating action by reducing friction between lids and cornea surface. Because of the peculiar chemical physical characteristics of the polymer, when applied at the surface of the 3D HCE model, these tear substitutes interact with the epithelium structure at molecular level giving the interesting opportunity to modeling the corneal epithelium response during the molecular and morphological modifications induced by the dry condition. Any lubrificating or hydrating efficacy should be applied to the different mechanism observed.

## Methods

### Biologic model

The reconstructed HCE model was supplied by SkinEthic^®^ Laboratories (Nice, France). The model consisted of immortalized human corneal epithelial cells (IHCEC) cultured on an inert, permeable polycarbonate filter of 0.5 cm^2^ for 5 days at the air-liquid interface in a supplemented chemically defined medium (modified MCDB 153). The overall morphology of the HCE model was similar to that of human corneal epithelium, with a flattened superficial layer of non-keratinized cells. At the intermediate cell layer the cells displayed more lateral cytoplasmatic extensions than those in the basal layer, similar to the wing cells. The basal layer consisted of regular column cells. At the ultrastructural level, the basal membrane revealed mature hemidesmosomes and associated anchoring filaments that form the complex for attachment of the epithelium to the stroma in vivo. The resulting 3D construct showed the morphology of the stratified cellular organization of HCE which has previously been characterized for different relevant markers [11]. HCE tissues were shipped on day 5: upon arrival they were aseptically removed and placed in a 6-well culture plate (Falcon; VWR, Milan, Italy) with 1 ml of chemically-defined maintenance medium supplied by SkinEthic (changed every 24 h). Different batches of HCE were used, with an average thickness of 70 μm (as reported in the quality data sheet for each batch). For each product and assay 2 HCE have been used.

### PART 1: Experimentally induced in vitro dry eye on HCE model (EDEV)

HCE tissues were placed under controlled environmental conditions to mimic dryness (<40% relative humidity, 40 °C±5 °C temperature and 5% CO_2_). Tissues were investigated for different parameters (cell viability, histology, and mRNA expression of selected genes) at 24 h, 48 h, and 72 h after establishment of dry eye conditions. These tissues are coded as EDEV-HCE.

#### AlamarBlue assay

AlamarBlue (Sigma-Aldrich, Milan, Italy) is a redox indicator dye that produces a colorimetric change in response to metabolic activity. In viable cells AlamarBlue is reduced by cytochromes, NADPH, NADH, FADH, and FMNH. Unlike other markers (e.g., MTT and Trypan Blue) AlamarBlue does not interfere with cell viability. The absorption spectra of AlamarBlue changes from the oxidized form (blue) to the reduced form (red). 10% AlamarBlue was added directly to the medium of the tissue models cultivated in standard or controlled EDEV conditions and then incubated at 37 °C. The relationship between the two spectra was determined by measuring the absorbance at 570 and 600 nm. The percentage of reduced AlamarBlue represented the quantity of viable cells.

At specific time points (24 h, 48 h, and 72 h), the % reduction (survival rate) of AlamarBlue was calculated relative to non-treated tissues using the following formula (Alamar Assay Booklet from Biosource):

$$\text{\% reduction = }\frac{(\varepsilon \text{ox})\lambda_{\text{2}}*\text{A}\lambda_{\text{1}}-(\varepsilon \text{ox}){\text{l}}_{\text{1}}*\text{A}\lambda_{\text{2}}\text{ treated sample }\left(\text{EDEV}\right)}{(\varepsilon \text{ox}){\text{l}}_{\text{2}}*\text{A}{}^{\circ}\lambda_{\text{1}}-(\varepsilon \text{ox}){\text{l}}_{\text{1}}*\text{A}{}^{\circ}\lambda_{\text{2}}\text{ non treated tissue }\left(\text{positive control}\right)}*100$$

where λ_1_=570 nm and λ_2_=600 nm.

(εox)λ_1_=80,586 (molar extinction coefficient of Alamar Blue (oxidised) at wavelength 570 nm)
(εox)λ_2_=117,216 molar extinction coefficient of Alamar Blue (oxidised) at wave lenght 600 nm)
Aλ_1_=absorption of the sample at 570 nm
Aλ_2_=absorption of the sample at 600 nm
A°λ_1_=absorption of the positive control at 570 nm
A°λ_2_=absorption of the positive control at 600 nm

#### Histology

At the end of an experiment, a single HCE tissue was removed from the insert using a sharp scalpel and fixed in 10% formalin solution (HT501128). After embedding in paraffin, vertical sections (4 μm thick) were cut with a microtome and hematoxylin and eosin (H&E) stained following internal procedures. Histological samples were analyzed under a light microscope and the overall morphology of the epithelium and its modifications compared to untreated negative HCE controls.

#### Immunofluorescence

Vertical sections of frozen epithelium (7 µm thick) were cut with a cryotome (Leica CM 3050, Leica Microsystems AG, Wetzlar, Germany) and stored at –80 °C until staining. Sections were subjected to immunofluorescence with antibodies against MMP9 and MUC4. Sections were rinsed with 1% BSA-PBS and two different sets of primary antibodies added in 1% BSA-PBS: the mouse monoclonal anti-MMP9 clone GE-213 (1:800; Santa Cruz Biotechnology; DBA Italia, Segrate(MI), Italy) and the rabbit polyclonal anti-mucin 4 (1:50, Santa Cruz). A biotine/streptavidin detection system was used to amplify the signal. Biotinylated secondary antibodies bind to the primary antibody. FITC-streptavidin is then applied. After three washes in PBS the nuclei were labeled with propidium iodide and mounted in an anti-fade medium (Vectashield; Vector Laboratories; DBA Italia, Segrate(MI), Italy). Samples were viewed under a fluorescence microscope (Leica DMLB; Leica Microsystem, Wetzler, Germany, with a CCD Sony DXC 390P;).

#### Evaluation of microvilli and mucins with scanning electron microscopy

HCE Samples were fixed in 2.5% glutaraldehyde in 0.1 M phosphate buffer for 2 h at 4 °C. Slides were washed three times for 5 min in 0.1M phosphate buffer and then placed in 1% OsO_4_ in 0.1 M phosphate buffer. Samples were dehydrated through a graded series of ethanol and critical-point-dried in a CO_2_ liquid Bemar SPC 1500 (Bomar Co, Tacoma, WA) apparatus. Specimens were mounted on aluminum stubs with silver-conducting paint, sputtered with a thin (20 nm) film of gold, and observed with a ESEM QUANTA 200 (FEI, Eindhoven, The Netherlands).

#### Transcriptional study of mRNA (qRT–PCR)

At each treatment time point, total RNA was extracted from HCE using the RNAqueous kit according to the manufacturer’s protocol (Ambion-Applied Biosystems, Monza, Italy). The cDNAs were then synthesized using 2 µg of RNA template in a 20 µl reaction using the High-Capacity cDNA Reverse Transcription Kit (Ambion-Applied Biosystems). RNA (10 µl) was added to the master mix, which was subjected to reverse transcription in a thermal cycler (ABI PRISM 7500 Real Time PCR System; Ambion-Applied Biosystems) under the following conditions: 25 °C for 10 min, 37 °C for 60 min, 85 °C for 5 s. Real Time Polymerase Chain Reaction was subsequently performed in triplicate in a 25 µl final reaction volume using the Applied Biosystems ABI PRISM 7500 Real Time PCR System instrument with TaqMan^®^ assay (Ambion-Applied Biosystems). The cDNA was amplified using TaqMan Universal PCR Master Mix and TaqMan gene expression assay provided as a 20× Assay mix (Human *MMP9*: TaqMan probe MMP9 Hs00234579_m1; Human *TNFα*: TaqMan probe TNF Hs00174128_m1; Human *MUC4*: TaqMan probe MUC4 Hs00366414_m1; Human *DEFB4*: TaqMan probe DEFB4 Hs 00175474_m1; and Human *GAPDH* as the calibrator gene: Taqman probe GAPDH Hs99999905_m1). The PCR conditions were 95 °C for 10 min (AmpliTaq Gold DNA Polymerase Activation) followed by 40 amplification cycles (95 °C for 15 s then 60 °C for 1 min).

The Relative Quantification (RQ) minimum and RQ maximum define statistical boundaries for relative quantification, based on a 95%-specified RQ Min/Max confidence setting. A confidence setting of 95% means that the user can expect the true RQ value to fall within the RQ Min/Max range with a 95% confidence. The RQ Min/Max is calculated using the equation:

$${\text{2}}^{-\text{ (}\Delta \Delta \text{C}}{}_{\text{T(s,t)}}{}^{\pm \text{T x VAB}(\text{C}}{}_{\text{T(s,t)}}{}^{))}$$

where ΔΔC_T(s,t)_=ΔC_T(s,t)_ - ΔC_T(calibrator,t)_; s=sample name; t=target detector; T=student’s T value at the selected confidence setting using a degree of freedom that is associated with the test sample ΔC_T(s,t)_; and VAB is the Applied Biosystems’ variability function for calculating the variability of the test sample ΔC_T_ statistic.

Calculations of relative gene expression used relative differences in the threshold cycle C_T_ (the PCR cycle at which the fluorescence reaches a given value or threshold that is in the log-linear range of amplification). Samples containing higher levels of expression of a particular gene reach the threshold value at lower cycle numbers during PCR than samples containing lower levels of expression of the same gene. Because each cycle in the PCR reaction corresponds to a twofold increase in PCR product, a difference of one in threshold cycle number represents a twofold difference in the expression of a particular gene or internal control target sequence and can be considered as significant. Duplicate cultures were analyzed and the data were represented as Relative Quantification data (RQ) ± standard deviation. The PCR instrument’s integrated software set a confidence level of 95% (i.e., for a true range of biologic significance).

### PART 2: Products, controls and treatments

In the second part of the study a serie of commercially available tear substitutes, were tested by Real Time PCR for their efficacy and capability to prevent desiccation of HCE tissue in our EDEV model. Scanning electron microscopy (SEM) and histological analysis were performed at selected treatments time points.

The following treatments (all routinely used for the long-term treatment of dry eye symptoms) were tested: sodium hyaluronate 0.2% (Hyalistil®; SIFI, Catania, Italy); TS-Polysaccharide (TSP 0.5%®); sodium hyaluronate 0.2% and TS-Polysaccharide 0.2% (Xiloial®; Farmigea, Pisa Italy) ; and carbossimethylcellulose (Optive®; Allergan, Irvine, CA). A dexamethasone preparation 0.15% (Etacortilen®; SIFI, Catania, Italy) was also assessed. According to the chemical and physical preservative systems (Table 1), a different cytotoxicity should be expected. The products have been previously tested for their compatibility and for 24 h application any cytotoxic effect has been quantified (data not shown).

**Table 1:** Identification of commercially available tear substitutes.

**Identification code**	**Active ingredient**	**Preservation system**
Etacortilen®	DEXAMETHASONE 0.15%	UNPRESERVED
Hyalistil®	SODIUM HYALURONATE 0.2%	THIOMERSAL
Xiloial®	SODIUM HYALURONATE 0.2% TS-Polisaccaride 0.2%	UNPRESERVED
TSP 0,5%®	TS-Polisaccaride 0,5%	UNPRESERVED
Optive®	CARBOSSIMETHYLCELLULOSE 0.5% Glycerin 0.9%	PURITE®

**Figure 1:**
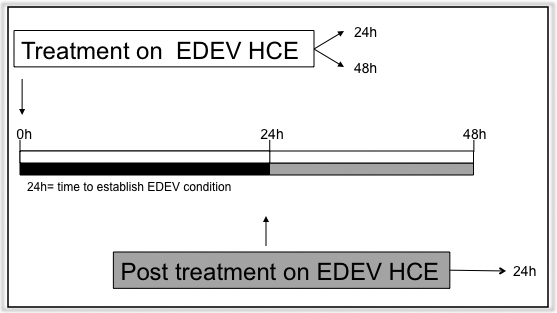
Schematic representation of protocol of EDEV induction and products treatment. EDEV: Experimentally induced in vitro dry eye.

Tear substitutes were applied via two different protocols (Figure 1). In the first procedure (treatment during EDEV induction), 50 μl of test product was directly applied and gently spread over the whole epithelium surface at the same time as induction of dry eye conditions. Tissues were maintained for 24 h and 48 h to determine the efficacy of the test products in counteracting the establishment of EDEV. In the second procedure (post-treatment on established EDEV), 50 μl of test product was applied to tissues previously maintained for 24 h under EDEV conditions; tissues were then followed for further 24 h to determine the ability of the test product to restore the normal eye condition. As a negative control, HCE were maintained under standard conditions (90% humidity, 37 °C and 5% CO_2_) and treated with saline solution for its neutral effect on the epithelium surface; these tissues are coded as CONTROL-HCE. Finally, untreated HCE tissues maintained in the controlled EDEV setting (<40% humidity, 40 °C, and 5% CO_2_) were used as a positive control (EDEV-HCE).

### Statistical analysis

Statistical comparisons of controls with treatment groups for qRT–PCR data were performed using the Student *t*-test.

## Results

### PART 1

#### Cell viability

**Figure 2:**
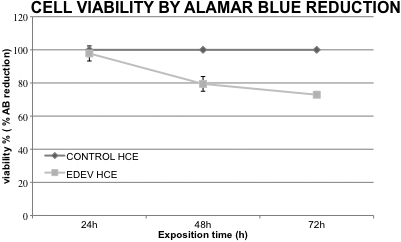
Reduction of AlamarBlue (%) resulting in % viability in EDEV-HCE compared to the CONTROL-HCE at 24 h, 48 h, and 72 h. The vertical lines represent the standard deviation (SD) between two different tissues. HCE: human corneal epithelium.

The viability of HCE tissue was evaluated by AlamarBlue (AB) at 24, 48, and 72 h after dry eye induction and compared with control HCE. The test has been performed on duplicate cultures. As shown in Figure 2, after the maximum exposure time (72 h), the survival of HCE in EDEV conditions was still quite good (70% cellular viability) compared with the CONTROL-HCE (cellular viability 100%).

#### mRNA quantification of *MMP9*, *MUC4*, *DEFB4*, and *TNF-α*

**Figure 3:**
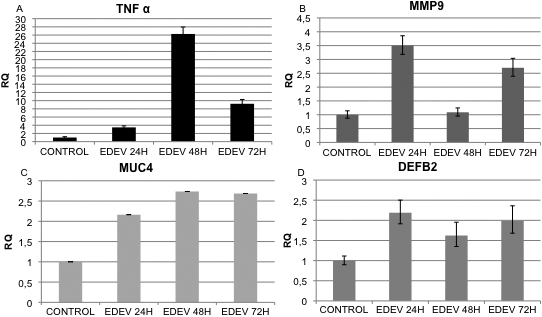
Set-up of EDEV model. Real-time PCR analysis showing the relative changes of *TNF-α* (**A**), *MMP9* (**B**), *MUC4* (**C**), and *DEFB2* (**D**) mRNA Control and EDEV HCE. Values are means± SD of two different HCE tissues.

Duplicate cultures for the gene expression study have been used. The transcriptional study by qRT–PCR revealed that expression of the inflammatory cytokine *TNF-α* was increased at 24 h following the induction of EDEV conditions. *TNF-α* expression was maximal at 48 h and remained significantly higher than levels in untreated CONTROL-HCE tissues until 72 h post-EDEV induction (Figure 3A). Overexpression of *MMP9* was observed at 24 h and 72 h following EDEV induction (Figure 3B). Consistently high levels of *MUC4* expression were observed in EDEV-HCE tissues at all time points analyzed (Figure 3C). A moderate increase in the expression of *DEFB2* in EDEV-HCE was observed at 24 h and 72 h after dry eye induction (Figure 3D).

The overexpression of *MMP9* and *MUC4* was expected under EDEV conditions. Previous studies have shown that *MMP-9* correlates with increased degeneration of the extracellular matrix due to inflammation and tissue sufferance. Upregulation of *MUC4* occurs as an epithelial defense mechanism in response to loss of tissue integrity and microvilli disappearance [24].

#### Histological analysis

**Figure 4:**
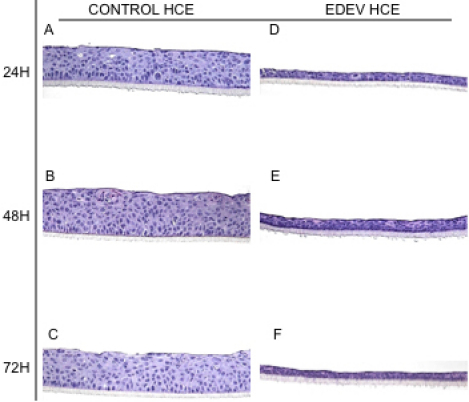
Histomorphological analysis of 3D-HCE after 24 h, 48 h, and 72 h cultivated in control condition (**A**, **B**, **C**) and in controlled EDEV condition (**D**, **E**, **F**). Magnification 20×.

Figure 4 shows the overall morphology viewed under a light microscope of CONTROL-HCE and EDEV-HCE tissue at 24 h, 48 h, and 72 h. The resolution of the pictures is the maximum allowed to see the morphology of cells: in case of reduced hydration the resolution of the images is less clear. CONTROL-HCE tissue morphology was perfectly preserved at all three time points studied (Figure 4A-C): a flattened layer of non keratinized superficial cells, an intermediate cell layer (with cells displaying lateral cytoplasmic extensions similar to the wing cells), and the basal layer of regular column cuboidal cells were all clearly visible. A small and normal increase in tissue thickness was observed over time.

A dramatic reduction in the thickness of the epithelium was observed in EDEV-HCE compared to CONTROL-HCE tissue (Figure 4D-F). It is important to highlight that the epithelium in EDEV-HCE was viable, as assessed by AlamarBlue at 24 h (100% viability), 48 h (80% viability), and 72 h (70% viability). The reduction in thickness appears to be related to loss of water due to the severe dry experimental conditions. Therefore, we concluded that low humidity and high temperature appear to reproduce the dry environmental conditions that cause ocular discomfort and inflammation.

#### Immunofluorescence analysis for MMP9 and MUC4

**Figure 5:**
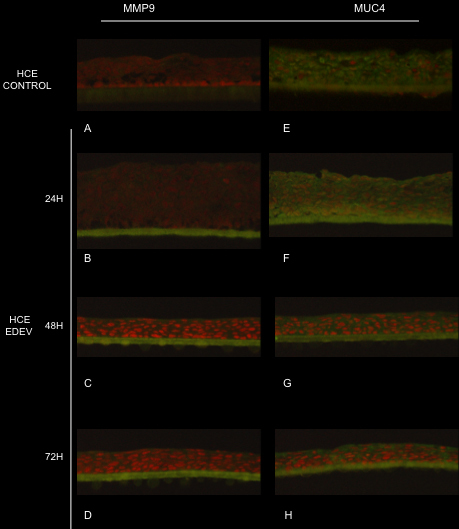
Immunofluorescence staining of MMP9 (**A**, **B**, **C**, **D**) and MUC4 (**E**, **F**, **G**, **H**) in control and EDEV HCE at 24 h, 48 h, and 72 h. MMP9 and MUC4 are stained in green and red nuclei are stained with propidium iodide.

Immunofluorescence analysis of EDEV-HCE at 24 h following induction of EDEV under controlled conditions showed diffuse but weak MMP9 staining (green) in almost all layers of the epithelium (red nuclei stained with propidium iodide) that was absent in CONTROL-HCE tissue. The MMP9 signal was increased at 48 h and 72 h after induction of dry eye condition. At 24 h, MUC4 was expressed constitutively in CONTROL-HCE in almost all cellular layers, and strong MUC4 staining was observed in EDEV-HCE (Figure 5). At 48 h and 72 h, the protein revealed in EDEV-HCE a preferential apical localization. The CONTROL-HCE tissues in Figure 5A and 5E showed respectively MMP9 and MUC4 expression after 24h. CONTROL-HCE at 48h and 72h were not illustrated because any difference from 24h control has been noted.

#### Microvilli analysis by scanning electron microscopy

**Figure 6:**
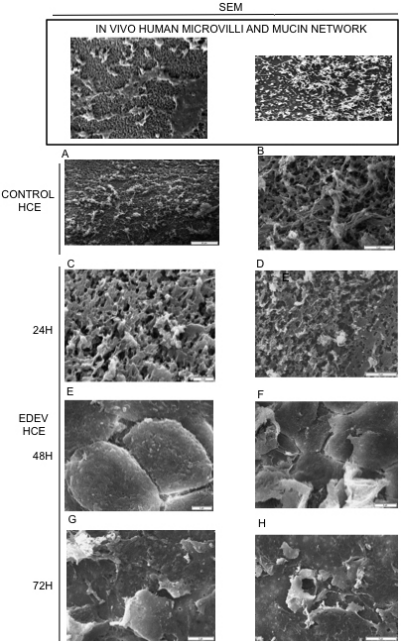
SEM images of human corneal microvilli and mucin network and in standard and EDEV HCE. Scanning electron microscopy showing HCE epithelium in control HCE condition (**A**, **B** at 20 and 5 μm of magnification) and in EDEV setting at 24 h (**C**, **D**), 48 h (**E**, **F**), and 72 h (**G**, **H**). The microvilli in **C**-**H** are identified at a different magnification (2–5 μm). The in vivo impression cytology with SEM on humans is illustrated in the box on the top of the Figure (microvilli on the left and mucin network on the right). The reproduction of the images are authorized by Professor Del Prete (University of Napoli).

SEM analysis revealed that CONTROL-HCE tissue was rich in microvilli and surprisingly close to the human model. Figure 6A,B show the epithelium surface at different magnifications. A progressive reduction in the number of microvilli was observed following induction of EDEV (Figure 6C-H). At 24 h post-induction, the number of microvilli was reduced (Figure 6C,D); microvilli were no longer present at 48 h and 72 h, and the desiccating stress was apparent on the epithelium surface. In the EDEV condition the tissue integrity was no longer observed (Figure 6E-H). Every time point was shown at two different magnification (2 and 5μm) to better discriminate the morphology of the cells.

Considering the results of PART I for all the parameter tested and our encouraging gene transcription results in line with clinical findings showing increased expression of *MMP9*, *TNF-α*, *MUC4*, and *DEFB2* [18,19,22], the EDEV-HCE model was subsequently used to assess the efficacy of tear substitutes.

### PART 2

#### Efficacy of tear substitutes in counteracting or repairing dryness in the EDEV-HCE model

As the experimentally established EDEV model could successfully mimic the features of a dry ocular surface within 24 h of EDEV induction (shown in PART 1), we tested the effects of various commercially available artificial tears with proven efficacy for the induced dry eye condition.

#### Real-time PCR analysis

#### Application of tear substitutes during EDEV induction

**Figure 7:**
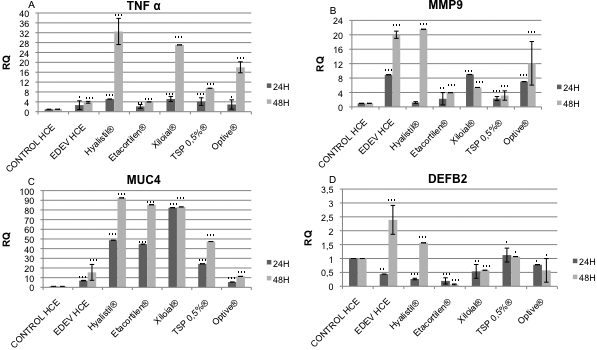
Results of EDEV biomarkers gene expression study. Real-time PCR analysis showing the relative changes of *TNF-α* (**A**), *MMP9* (**B**), *MUC4* (**C**), and *DEFB2* (**D**) mRNA after 24 h and 48 h treatment with different tear substitutes simultaneously the induction of EDEV. A twofold change of Relative Quantification (RQ) is usually considered significant in comparison to the calibrator (CONTROL-HCE at each time point). A two-tailed Student’s *t*-test was used to determine statistical significance for real-time PCR. Data were considered significant at p<0.05 (*), p<0.01 (**), and p<0.001 (***).

All tear substitutes had a significant stronger effect on *TNF-α* mRNA increase after 48 h of treatment compared to 24 h. After 24 h of treatment, all of the tear substitutes tested induced an overexpression of *TNF-α*. Etacortilen^®^, a well known anti-inflammatory drug, was the only agent able to control and block the expression of the inflammatory genes to the same RQ values as those observed in the EDEV-HCE (Figure 7A). The upregulation of *MMP9* observed at 24 h in the EDEV model was controlled by treatment with all tear substitutes except for Xiloial^®^ and Optive^®^. At 48 h all the tears except Hyalistil^®^ were able to counteract the increased expression of *MMP9* (Figure 7B).

Higher levels of *MUC4* mRNA were observed after 24 h of Xiloial^®^ treatment compared to the other tear substitutes and EDEV-HCE (Figure 7C). However all products except Optive^®^ after 24 h and 48 h induced a significant upregulation of *MUC4* expression compared to EDEV-HCE. Membrane-associated mucins have a role as a protective barrier gene. Increased expression of *MUC-4* is associated to a positive adaptive mechanism to counteract the atrophy and the loss of lipidic film.

No significant differences were found in the expression of *DEFB2* compared to EDEV-HCE after 24 h of treatment with Hyalistil^®^, Etacortilen^®^, and Xiloial^®^. TSP 0.5%^®^ and Optive^®^ restored the Control HCE value of *DEFB2* (Figure 7D). After 48 h, all of the tear substitutes had counteracted the increase in *DEFB2* expression observed in EDEV-HCE (Figure 7D).

#### Application of tear substitutes on established EDEV-HCE

As shown in Figure 1, tear substitutes were applied to tissues previously maintained for 24 h under EDEV conditions; established EDEV-HCE tissues were then followed for further 24 h to assess polymers activity on the expression of the selected biomarkers and on the microvilli structure.

**Figure 8:**
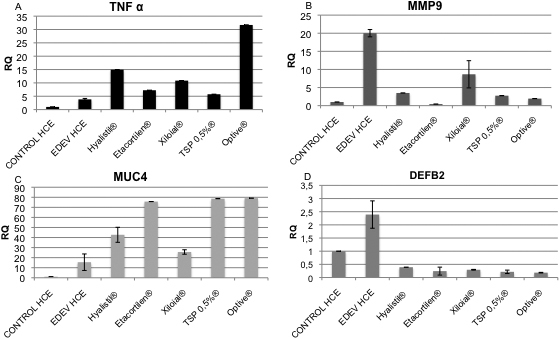
Results of EDEV biomarkers gene expression study. Real-time PCR analysis showing the relative changes of *TNF-α* (**A**), *MMP9* (**B**), *MUC4* (**C**), and *DEFB2* (**D**) mRNA after 24 h treatment on established EDEV model. A twofold change of Relative Quantification (RQ) is usually considered significant in comparison to the calibrator (CONTROL-HCE at each time point). A two-tailed Student’s *t*-test was used to determine statistical significance for real-time PCR. All the data presented in this graphic were significant at p<0.001.

In the established EDEV-HCE model, *TNF-α* expression was upregulated following treatment with Optive^®^, Xiloial^®^, and Hyalistil^®^. On the contrary Etacortilen^®^ and TSP 0.5%^®^ did not induce an increase of *TNF-α*, suggesting an anti-inflammatory efficacy (Figure 8A). All of the products applied showed efficacy in controlling *MMP9* expression. A decrease of *MMP9* expression in comparison to EDEV alone was observed (Figure 8B). All tear substitutes increased *MUC4* mRNA expression relative to EDEV-HCE (Figure 8C). A significant decrease in *DEFB2* expression was also observed following treatment with all the tears substitutes compared to EDEV-HCE (Figure 8D).

#### Histologic analysis at 24 h

**Figure 9:**
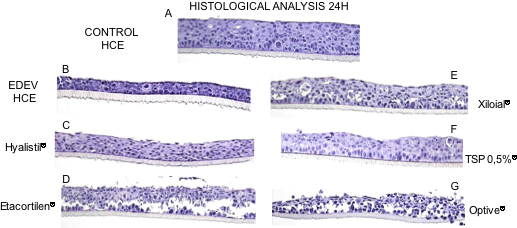
Histological analysis. **A**: Normal morphology of control HCE with saline solution. **B**: Epithelial thickness reduced in EDEV-HCE. **C**: Hyalistil^®^; **D**: Etacortilen^®^; **E**: Xiloial^®^; **F**: TSP 0,5%^®^; **G**: Optive^®^. Magnification 20×.

Figure 9 shows the morphology of HCE after treatment with various tear substitutes. Epithelial thickness was reduced in EDEV-HCE compared to CONTROL-HCE at 24 h (Figure 9A). After 24 h simultaneous treatment with EDEV induction, Hyalistil^®^, Xiloial^®^, and TSP 0.5% restored the initial morphology of HCE, which resembled the standard conditions (Figure 9C,E,F). Etacortilen^®^ and Optive^®^ induced epithelial damage; the integrity of the epithelium was partially lost (Figure 9D-G).

#### SEM analysis at 24 h

**Figure 10:**
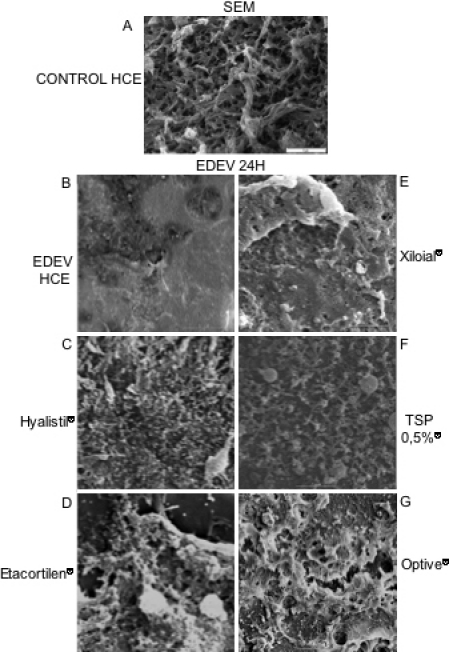
Images of microvilli in in vitro reconstituted corneal epithelium. Scanning electron microscope images showing Control (**A**) and EDEV-HCE tissue induced for 24 h (**B**) with treatments with different tear substitutes (**C**-Hyalistil^®^; **D**-Etacortilen^®^; **E**-Xiloial^®^; **F**-TSP 0,5%^®^; **G**-Optive^®^). Two μm magnification.

SEM images showing the overall morphology after treatment of HCE are shown in Figure 10. In EDEV-HCE after 24 h, the microvilli almost completely disappeared with the breakdown of epithelial cells (Figure 10B). Partial restoration of microvilli density was observed with some of the products (Figure 10C-G) compared to the CONTROL-HCE (Figure 10A); in particular, the morphology of microvillial cells conserved for TSP 0.5%^®^, Xiloial^®^ and Hyalistil^®^.

## Discussion

The present study investigated the biologic response to dry environmental cultivation conditions in a 3D model of HCE by monitoring the kinetics of a biologically relevant gene signature over a 72 h time period. By using this dynamic approach it was possible to define the optimal duration of the dryness conditions to assess the activity of different polymers contained in topically-applied tear substitutes. This new EDEV model was induced by exposure of HCE to a low-humidity environment (<40%) and increased temperature (up to 40 °C). We showed that the time necessary to induce this experimental model and to mimic the characteristic features of the corneal epithelium in the DTS was 24 h; during this time cell viability was not affected as quantified by AlamarBlue Assay. The transcriptional study has shown the relevance of selected biomarkers and the tissue response to EDEV setting and product treatment confirmes the role of each gene as described in the literature.

The EDEV-HCE model is characterized by increase in *MUC4*, *MMP9*, *TNF-α*, and *DEFB2* expression.

Transcription studies on the gene signature for epithelial dryness confirmed previous clinical findings showing alterations in the carbohydrate component of mucins in dry eye, resulting in abrasive stress and epithelial damage [25]. MMP9 was increased in the tear fluid of patients with dry eye [17] and increased production and activation of pro-inflammatory cytokines (TNF-α and IL1-α) have been observed at the stressed ocular surface in dry eye [18,19]. Furthermore DEFB2 levels were increased in subjects with dry eye showing signs of ocular irritation [26].

The EDEV-HCE model stimulated the production of inflammatory factors (TNF-α and MMP-9). Higher levels of TNF-α were found at all time points studied (24 h, 48 h, and 72 h) compared with CONTROL-HCE tissue. These data are in line with the results described in other studies, which found increased concentrations of pro-inflammatory cytokines and chemokines (such as IL-6, IL-1, TNF-α) in the tear fluid [18,19]. In dry eye, increased production and activation of pro-inflammatory cytokines (interleukin IL-1 and TNF-α) and proteolytic enzymes produced by stressed ocular surface and glandular epithelial cells, as well the inflammatory cells that infiltrate these tissues, have been reported.

Increased levels of MMP9 were also present in EDEV-HCE at 24 h and 72 h following EDEV induction. Increased levels of proinflammatory cytokines and metalloproteinases have been observed on the ocular surface of patients with KCS [27]. Increased concentration and activity of MMPs in the tear fluid of patients with dry eye have previously been reported [17]. Furthermore, increased levels of *TNF-α* and *MMP9* mRNA were shown with real-time and gene array data in an induced dry eye model in the mouse [6]. TNF-α and IL-1 also stimulate the production of MMPs by epithelial and inflammatory cells [28]. MMP9 is the most important gelatinase present on the ocular surface and MMP9 levels appear to be higher in the tear fluids of patients with KCS and in particular in patients with ulceration. MMP-9 lyses a variety of different substrates including components of the corneal epithelial basement membrane and tight junction proteins (such as zonula occludens-1 [ZO-1] and occludin), compromising the integrity of the corneal epithelial barrier. Proteolitic cleveage of occludin accelerates loss of superficial corneal epithelium, thus increasing the permeability of corneal epithelium. An *MMP9* knockout mouse model confirmed that increased *MMP9* activity in response to experimental dry eye disrupts the corneal epithelial barrier [29]. *MMP-9* appears to play a physiologic role in regulating corneal epithelial desquamation. The increased *MMP-9* activity in KCS may be responsible in part for the deranged corneal epithelial barrier function, increased corneal epithelial desquamation, and corneal surface irregularity.

A twofold induction of *MUC4* mRNA occurred in EDEV-HCE. MUC4 is a membrane-bound mucin and is secreted by conjunctival and corneal epithelial cells [30,31]. *MUC4* upregulation found in EDEV-HCE model can be interpreted as an early marker that acts as a positive signal to stimulate the production of mucin in a situation in which mucin protein is lacking. Three types of mucins are present at the ocular surface. The large gel-forming mucin-5 AC (MUC5AC) is expressed by conjunctival goblet cells. Some cells of the lacrimal gland acini express the small soluble mucin MUC7. The corneal and conjunctival epithelia express the membrane-associated mucins (MUCs 1, 4, and 16) [4]. These mucins are mainly concentrated on the tips of the apical cells’ microplicae, forming a dense glycocalyx at the epithelial-tear film interface. However, they can also be released from the cell surface and are found in the tear film [20]. Traditionally, mucin function at the ocular surface has been ascribed to secreted gel-forming mucins acting as lubricating agents and clearing molecules. New evidence shows a role for cell surface-associated mucins in providing barrier function to corneal and conjunctival epithelia.

Cell surface-associated mucins form a thick cell surface glycocalyx that may extend up to 500 nm from the plasma membrane at the ocular surface. Cell surface-associated mucins provide dishadesive properties, boundary lubrication and prevent adhesion of facing cell surfaces (i.e., corneal epithelium and tarsal conjunctiva) during blinking or sleeping [25]. These mucins can contribute to the maintenance of the mucosal barrier integrity, preventing the penetration of extracellular molecules on ocular surface epithelia and maintaining epithelial protection, and therefore a healthy wet-surface phenotype. Recent studies on cell surface-associated mucins have shown that their carbohydrate component is altered in dry eye syndrome. Alteration of mucin O-glycosylation in dry eye compromises the ocular surface epithelial barrier and results in increased abrasive stress and epithelial damage. Ocular mucus protects against bacterial adherence to the corneal epithelium and alteration in mucus production promote bacterial adherence to the cornea [32].

The ocular surface in dry eye disease is compromised and therefore at risk for microbial infections. Subjects with dry eye may have an alternative mechanism to protect the ocular surface from infection. One possibility is that naturally occurring antimicrobial peptides, such as human β-defensins (hBDs), provide alternate means of defense for the compromised ocular surface in dry eye, thus reducing the risk of corneal and conjunctival infections [22]. Defensins are antimicrobial peptides that are involved in innate host defense. β-defensins are secreted by epithelial cells and are active in vitro against several different bacteria, fungi and enveloped viruses and are thought to perform their microbicidal functions by forming pores in microbial cell membranes. Although hBDs are primarily antimicrobial in nature, they can influence cellular activities such as proliferation, cytokine production, and histamine release by mast cells. Defensins also have chemotactic effects on mast cells, T-cells, dendritic cells, and monocytes and promote dendritic cell maturation. At least some of these functions appear to be mediated by receptors such as toll-like receptor-4, and CC-chemokine receptor-6. These other activities have led to the suggestion that defensins are a link between innate and adaptive immunity. It is possible that expression of hBD-2 plays a chemotactic role in mediating the increase in T-cell subpopulations observed in subjects with non-Sjögren’s dry eye. Further, hBD-2 stimulated histamine release by conjunctival mast cells may elicit signs and symptoms of ocular irritation. Therefore, while an increased expression of hBD-2 may be beneficial in terms of antimicrobial protection, it may also contribute to ocular surface damage observed in subjects with dry eye. The observed low but significant upregulation of beta-defensin 2 in the EDEV-HCE seems to be rather a defense mechanism consequent to the damage observed than an active mechanism of antimicrobial protection.

A gradual breakdown and disruption of epithelial integrity following EDEV induction was observed in our SEM experiments over the 72 h period. However, EDEV-HCE tissue viability and morphology was fairly well preserved, as indicated by the viability assay and histomorphologic analysis, which showed a reduction in EDEV-HCE tissue thickness.

Our results suggest that this new experimentally induced EDEV-HCE model can been used to test the efficacy of a series of polymers in commercially artificial tears in preventing or reducing the molecular and structural modifications characteristic of the DTS at corneal epithelium level. Dry eye symptoms are commonly treated with these eye drops; formulations are designed to interact with the mucus and aqueous layers of the tear film, and thanks to their composition replacing lost moisture and stabilizing the tear film.

The application of tears substitutes during EDEV induction allowed products discrimination for their efficacy in promoting the increase of MUC4 (Etacortilen^®^, Hyalistil^®^, Xiloial^®^, and TSP 0,5%^®^), associated to a positive adaptive and defense mechanism to fight the atrophy and the loss of lipidic film. Products were also discriminated for their efficacy in decreasing TNF-α (Etacortilen^®^) correlated to an antiinflammatory mechanism, in limiting the overexpression of MMP9 (Etacortilen^®^, Hyalistil^®^, and TSP 0,5%^®^ at 24 h and Etacortilen^®^, TSP 0,5%^®^, and Xiloial^®^ at 48 h) and in regulating the DEFB2 restoring the control HCE value (TSP 0,5%^®^at 24 h) and counteracting the increasing DEFB2 gene ((Etacortilen^®^, Hyalistil^®^, TSP 0,5%^®^, Xiloial^®^, and Optive^®^at 48 h) correlated to an anti-microbial effect on the ocular surface and protection from ocular damage.

In the post EDEV treatment, the efficacy in restoring the control HCE homeostasis has been quantified for the ability in increasing MUC4 (Etacortilen^®^, Hyalistil^®^, TSP 0,5%^®^, Xiloial^®^, and Optive^®^), in contrasting the increase of TNF-α (Etacortilen^®^ and TSP 0,5%^®^), in decreasing *MMP9* and *DEFB2* (Etacortilen^®^, Hyalistil^®^, TSP 0,5%^®^, Xiloial^®^, and Optive^®^).

This study has demonstrated that our EDEV-HCE model reproduce the morphological and molecular modifications to the corneal epithelium in the DTS suitable to test products activity in preclinical trials in both counteracting the induction of dryness conditions or restoring the homeostasis in an established EDEV-HCE model.

By using a dynamic approach, we were able to define a gene signature of DTS that could be predictive of corneal damage in vivo. The transcriptomic approach applied to 3D human tissues appears to be an encouraging method for gaining a deeper understanding of the DTS on corneal epithelium and for studying the epithelial response with non-invasive techniques. By using this new experimentally-induced EDEV model, it was possible to discriminate between different artificial tear products (with respect to the mechanism of action of polymers and others ingredients) suggesting that this may represent a promising tool for preclinical screening or for research studies on the molecular underlying mechanisms of the DTS.
